# A Practical Nomogram to Predict Early Death in Advanced Epithelial Ovarian Cancer

**DOI:** 10.3389/fonc.2021.655826

**Published:** 2021-03-19

**Authors:** Zixuan Song, Yangzi Zhou, Xue Bai, Dandan Zhang

**Affiliations:** ^1^Department of Obstetrics and Gynecology, Shengjing Hospital of China Medical University, Shenyang, China; ^2^Department of Health Management, Shengjing Hospital of China Medical University, Shenyang, China

**Keywords:** advanced epithelial ovarian cancer, nomograms, early death, SEER database, prognosis

## Abstract

**Background:** Ovarian cancer is a common gynecological malignancy, most of which is epithelial ovarian cancer (EOC). Advanced EOC is linked with a higher incidence of premature death. To date, no effective prognostic tools are available to evaluate the possibility of early death in patients with advanced EOC.

**Methods:** Advanced (FIGO stage III and IV) EOC patients who were enrolled in the Surveillance, Epidemiology, and End Results database between 2004 and 2015 were regarded as subjects and studied. We aimed to construct a nomogram that can deliver early death prognosis in patients with advanced EOC by identifying crucial independent factors using univariate and multivariate logistic regression analyses to help deliver accurate prognoses.

**Results:** In total, 13,403 patients with advanced EOC were included in this study. Three hundred ninety-seven out of a total of 9,379 FIGO stage III patients died early. There were 4,024 patients with FIGO stage IV, 414 of whom died early. Nomograms based on independent prognostic factors have the satisfactory predictive capability and clinical pragmatism. The internal validation feature of the nomogram demonstrated a high level of accuracy of the predicted death.

**Conclusions:** By analyzing data from a large cohort, a clinically convenient nomogram was established to predict premature death in advanced EOC. This tool can aid clinicians in screening patients who are at higher risk for tailoring treatment plans.

## Introduction

Ovarian tumor is the second most common malignancy in females, with a 5-year survival rate of only 48.6% (1, 2). Epithelial ovarian cancer (EOC) is the most prevalent type of ovarian tumor, which accounts for ~90% of all tumors (3). The four most common subtypes of EOCs are serous, endometrioid, clear cell, and mucous carcinoma (2). EOC is diagnosed with advanced disease in ~70% of the patients (4). Although debulking surgery combined with neoadjuvant or adjuvant platinum- or purple gold-based chemotherapy has become the standard treatment approach for patients with advanced EOC, survival rates for advanced ovarian cancer are still low, patients who are classified as stage III and IV under the Federation International of Gynecology and Obstetrics (FIGO) have the 5-year survival rates of only 42 and 26%, respectively (3, 5). In advanced EOC, a subset of patients dies within 3 months of diagnosis. For these patients, aggressive treatment did not significantly delay the survival of the patients, but reduced the quality of life of the patients. Exploring the related factors of early death can benefit clinicians as it distinguishes high-risk patients promptly and targeted palliative care, such as relieve cancer pain and other adverse symptoms, to improve the quality of life. To the best of our knowledge, very little attention has been paid to inspect factors associated with early death in advanced EOC. A premature mortality prediction model has become essential for patients with advanced EOC in order to guide oncologists to personalize treatments for patients.

The Surveillance, Epidemiology, and the End Results (SEER) database (https://seer.Cancer.gov/) have been widely used in the study of tumor pathogenesis and survival (6, 7). The SEER database registers ~34.6% of U.S. cancer patients and contains a wealth of clinical information. The SEER database had a larger number of patients compared to the single-center study. The present study evaluated the frequency of early death from advanced EOC recorded in the SEER database between 2004 and 2015 and established a prognostic model for early identification of high-risk patients.

## Materials and Methods

### Ethics Statement

The SEER database data does not need informed patient consent, and cancer is a reportable disease in every state in the United States. This study was consistent with the 1964 Helsinki Declaration and subsequent amendments or similar ethical standards.

### Patients

**Figure 1:**
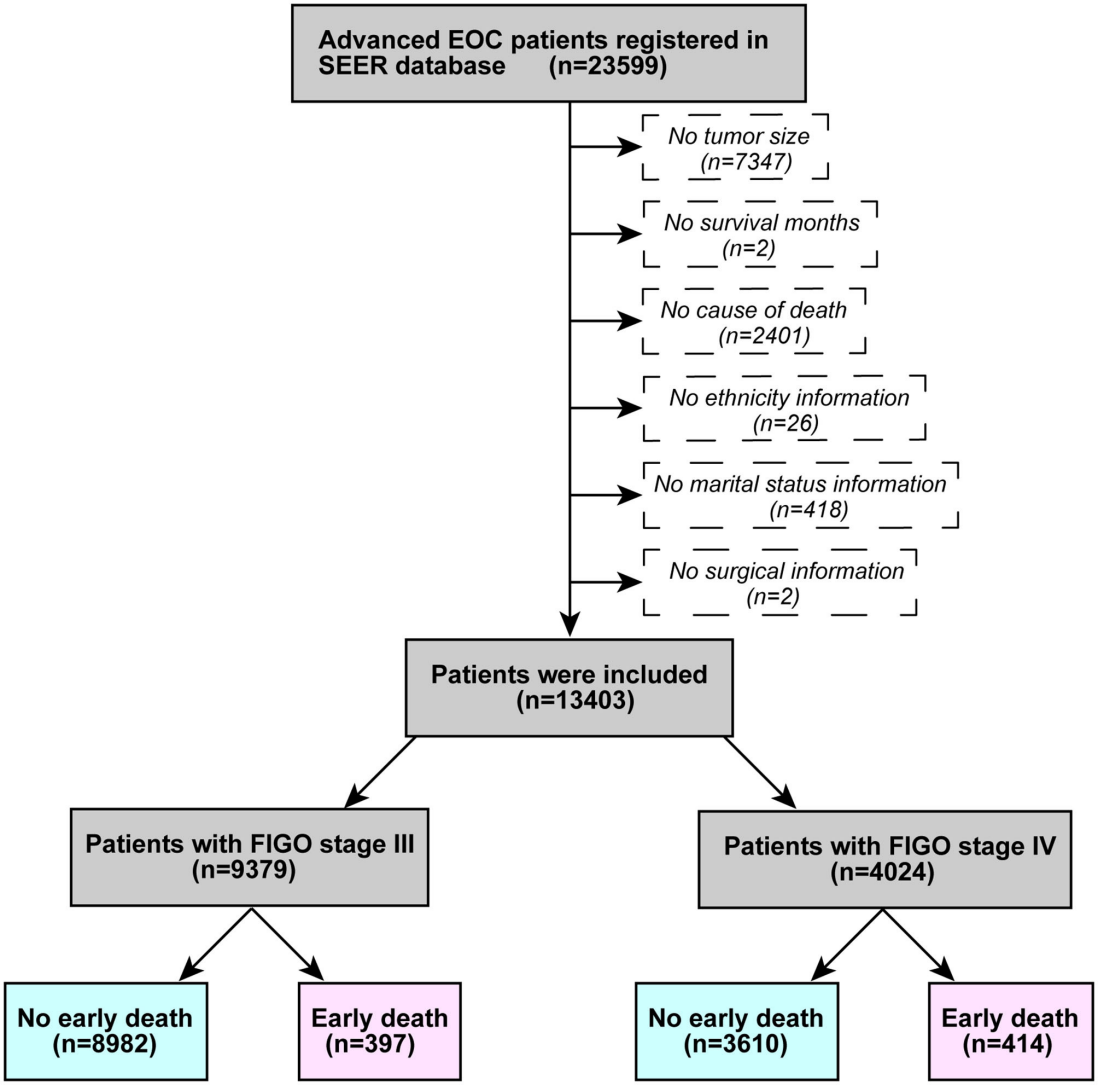
Patient selection flowchart.

Patient information was obtained using SEER*Stat (version 8.3.6.1). The inclusion criteria were as follows: (1) advanced ovarian epithelial neoplasm registered between 2004 and 2015 (FIGO stage III and IV), (2) Site code: C56.9, and (3) Histological code: 838/3-8482/3 (in accordance with the International Classification of Tumor Diseases, Third Edition (ICD-O-3). The excluding principles were: (1) lack of tumor size information; (2) no survival months recorded; (3) no cause of death; (4) no ethnicity; (5) no marital status; and (6) no surgical information. Figure 1 illustrates a flowchart of patient selection. According to past research (8, 9), death within 3 months of initial diagnosis was defined as early death.

### Data Collection

Information on patients with advanced ovarian epithelial tumors was extracted from the SEER database. (1) Demographic information including race, age, insurance status, and marital status (2) Clinical features: histological subclassification, histological grading, laterality, tumor size, metastasis location, surgical information, radiotherapy information, and chemotherapy information. (3) Main results: early death tends to occur in FIGO stage III and IV patients (≤ 3 months).

### Statistical Analysis

**Figure 2:**
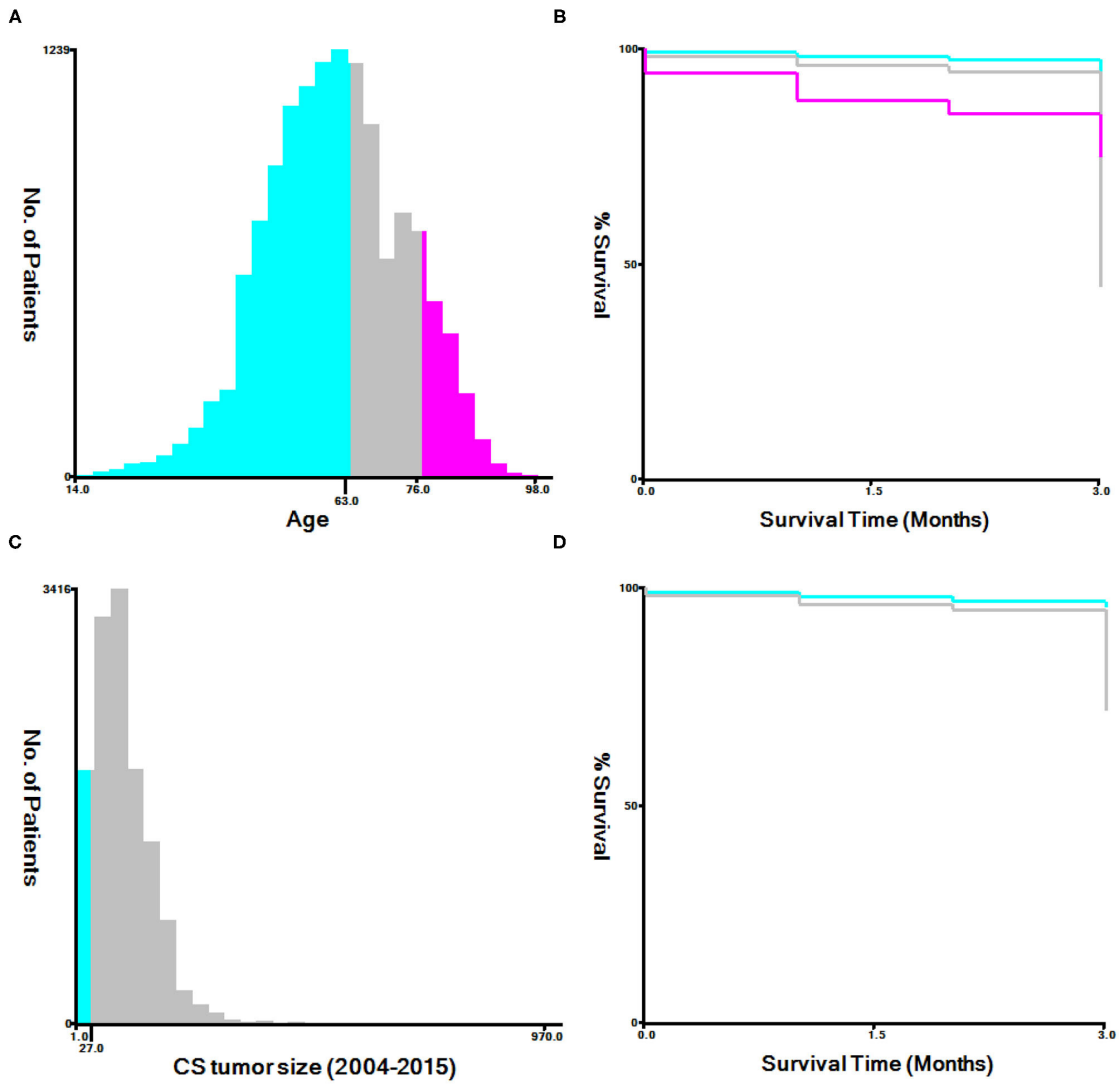
The appropriate age and tumor size cutoff values. **(A,B)**: The appropriate cutoff values of age were 63 and 76 years; **(C,D)**: The appropriate cutoff values of tumor size were 27 mm.

Using the X-tile software, the optimal cutoff values for age and tumor size were examined (10). The best cutoff values were 63 years and 76 years of age, and 27 mm for tumor size (Figure 2). All data was using Rsize. R Version 4.0.2 (The R Development Core Team, Vienna, Austria, http://www.r-project.org) to analyze in the RStudio environment. The statistical significance level was set at *P* < 0.05. Univariate and multivariate logistic regression analyses were both completed on the data gathered in order to examine factors linked with premature mortality. Constructed a nomogram based on the significant variables in multivariate logistic regression model. The receiver operating characteristic (ROC) curve was plotted to evaluate the performance of the nomogram (11). The R-language DCA package was utilized to assess the clinical impact of the nomogram by the decision curve analysis (DCA) (12). Bootstrapping (1,000 re-samplings) was used for internal validation, comparison between the validation model and original data was made using consistency statistics (C statistics) (13) and Brier score (14) to estimate the accuracy of the nomogram.

## Results

### Characteristics of Patients

A total of 23,599 patients with advanced ovarian epithelial tumors met the inclusion criteria, and 13,403 patients were eventually included in this study. There were 9,379 patients who were FIGO stage III, including 397 early deaths and 4,024 FIGO stage IV patients, including 414 early deaths. Table 1 demonstrates the patients characteristics.

**Table 1:** Characteristics with advanced epithelial ovarian cancer patients.

**Characteristic**	**FIGO stage III**		**FIGO stage IV**	
	**Non early death** **(*N* = 8,982)**	**Early death (*N* = 397)**	**Non early death** **(*N* = 3,610)**	**Early death (*N* = 414)**
**Age (years)**				
≤ 63	5,422 (60%)	100 (25%)	2,074 (57%)	153 (37%)
64–76	2,635 (29%)	139 (35%)	1,194 (33%)	137 (33%)
≥77	925 (10%)	158 (40%)	342 (9.5%)	124 (30%)
**Race**				
White	7,667 (85%)	340 (86%)	3,021 (84%)	327 (79%)
Black	549 (6.1%)	38 (9.6%)	279 (7.7%)	49 (12%)
Others	766 (8.5%)	19 (4.8%)	310 (8.6%)	38 (9.2%)
**Insurance**				
No	241 (2.7%)	8 (2.0%)	99 (2.7%)	9 (2.2%)
Yes	6,795 (76%)	297 (75%)	2,752 (76%)	298 (72%)
Unknown	1,946 (22%)	92 (23%)	759 (21%)	107 (26%)
**Marital status**				
Single	1,573 (18%)	67 (17%)	673 (19%)	87 (21%)
Married	5,273 (59%)	146 (37%)	2,002 (55%)	179 (43%)
Divorced or Separated	1,058 (12%)	58 (15%)	456 (13%)	51 (12%)
Widowed	1,078 (12%)	126 (32%)	479 (13%)	97 (23%)
**Histological subtype**				
Endometrioid carcinoma	603 (6.7%)	24 (6.0%)	164 (4.5%)	37 (8.9%)
Serous cystadenocarcinoma	7,703 (86%)	304 (77%)	3,181 (88%)	299 (72%)
Mucinous cystadenocarcinoma	226 (2.5%)	37 (9.3%)	110 (3.0%)	53 (13%)
Clear cell adenocarcinoma	450 (5.0%)	32 (8.1%)	155 (4.3%)	25 (6.0%)
**Grade**				
Grade I	344 (3.8%)	12 (3.0%)	78 (2.2%)	11 (2.7%)
Grade II	1,071 (12%)	42 (11%)	342 (9.5%)	35 (8.5%)
Grade III	3,977 (44%)	166 (42%)	1,567 (43%)	163 (39%)
Grade IV	2,544 (28%)	81 (20%)	984 (27%)	80 (19%)
Unkown	1,046 (12%)	96 (24%)	639 (18%)	125 (30%)
**Laterality**				
Only one side	3,679 (41%)	197 (50%)	1,382 (38%)	218 (53%)
Both sides	5,177 (58%)	179 (45%)	2,108 (58%)	164 (40%)
Unkown	126 (1.4%)	21 (5.3%)	120 (3.3%)	32 (7.7%)
**Tumor size (mm)**				
≤ 27	1,034 (12%)	32 (8.1%)	462 (13%)	25 (6.0%)
≥28	7,948 (88%)	365 (92%)	3,148 (87%)	389 (94%)
**Bone metastases**				
No	–	–	1,941 (54%)	197 (48%)
Yes	–	–	29 (0.8%)	5 (1.2%)
Unknown	–	–	1,640 (45%)	212 (51%)
**Brain metastases**				
No	–	–	1,954 (54%)	199 (48%)
Yes	–	–	4 (0.1%)	2 (0.5%)
Unknown	–	–	1,652 (46%)	213 (51%)
**Liver metastases**				
No	–	–	1,508 (42%)	137 (33%)
Yes	–	–	456 (13%)	62 (15%)
Unknown	–	–	1,646 (46%)	215 (52%)
**Lung metastases**				
No	–	–	1,627 (45%)	143 (35%)
Yes	–	–	343 (9.5%)	55 (13%)
Unknown	–	–	1,640 (45%)	216 (52%)
**CA125**				
Negative	331 (3.7%)	6 (1.5%)	90 (2.5%)	9 (2.2%)
Positive	7,257 (81%)	300 (76%)	3,034 (84%)	326 (79%)
Unkown	1,394 (16%)	91 (23%)	486 (13%)	79 (19%)
**Surgery**				
No surgery	139 (1.5%)	74 (19%)	201 (5.6%)	123 (30%)
Local resection	105 (1.2%)	11 (2.8%)	57 (1.6%)	23 (5.6%)
Debulking or pelvic exenteration	8,723 (97%)	311 (78%)	3,345 (93%)	266 (64%)
Surgery but the specific operation unknown	15 (0.2%)	1 (0.3%)	7 (0.2%)	2 (0.5%)
**Radiation**				
No	8,881 (99%)	394 (99%)	3,549 (98%)	410 (99%)
Yes	101 (1.1%)	3 (0.8%)	61 (1.7%)	4 (1.0%)
**Chemotherapy**				
No	1,117 (12%)	275 (69%)	430 (12%)	269 (65%)
Yes	7,865 (88%)	122 (31%)	3,180 (88%)	145 (35%)

*Statistics presented: n (%), FIGO, Federation International of Gynecology and Obstetrics*.

### Risk Factor Analysis for Early Death

The univariate and multivariate logistic regression analyses of early mortality in patients with advanced EOC are depicted in Tables 2, 3. Univariate analysis indicated that FIGO stage III patients and those who were older, of black race, widowed, and those who had mucinous cystadenocarcinoma, unilateral, large tumor, CA125 positive, non-operative, or non-chemotherapy patients had were at increased risk of premature mortality. In addition to the aforementioned risk factors, except for CA125 positivity, liver and lung metastases were also associated with increased risk of early death in FIGO stage IV patients. It was demonstrated through multivariate analysis that FIGO stage III, older, divorced/separated patients, as well as those with mucinous cystadenocarcinoma, CA125 positive, non-operative, or non-chemotherapy had an elevated risk of premature death. With regards to FIGO stage IV patients, elderly patients, and those with mucinous cystadenocarcinoma, liver metastases, lung metastases, and non-operative or non-chemotherapy, an increased risk of premature mortality was also observed.

**Table 2:** The univariable logistic regression analysis of early death in patients with advanced epithelial ovarian cancer.

**Characteristic**	**FIGO Stage III**			**FIGO Stage IV**		
	**OR**	**95% CI**	***P*-value**	**OR**	**95% CI**	***P*-value**
**Age (years)**						
≤ 63	Ref			Ref		
64–76	2.86	2.21, 3.72	<0.001^*^	1.56	1.22, 1.98	<0.001^*^
≥77	9.26	7.15, 12.0	<0.001^*^	4.91	3.78, 6.39	<0.001^*^
**Race**						
White	Ref			Ref		
Black	1.56	1.09, 2.18	0.012^*^	1.62	1.16, 2.22	0.003^*^
Others	0.56	0.34, 0.87	0.015^*^	1.13	0.78, 1.60	0.493
**Insurance**						
No	Ref			Ref		
Yes	1.32	0.69, 2.93	0.450	1.19	0.63, 2.56	0.621
Unknown	1.42	0.73, 3.22	0.346	1.55	0.80, 3.38	0.227
**Marital status**						
Single	Ref			Ref		
Married	0.65	0.49, 0.88	0.004^*^	0.69	0.53, 0.91	0.008^*^
Divorced or Separated	1.29	0.90, 1.84	0.170	0.87	0.60, 1.24	0.437
Widowed	2.74	2.03, 3.75	<0.001^*^	1.57	1.15, 2.14	0.005^*^
**Histological subtype**						
Endometrioid carcinoma	Ref			Ref		
Serous cystadenocarcinoma	0.99	0.66, 1.55	0.969	0.42	0.29, 0.61	<0.001^*^
Mucinous cystadenocarcinoma	4.11	2.42, 7.11	<0.001^*^	2.14	1.32, 3.49	0.002^*^
Clear cell adenocarcinoma	1.79	1.04, 3.10	0.036^*^	0.71	0.41, 1.24	0.234
**Grade**						
Grade I	Ref			Ref		
Grade II	1.12	0.60, 2.25	0.725	0.73	0.36, 1.56	0.383
Grade III	1.20	0.69, 2.29	0.555	0.74	0.40, 1.49	0.360
Grade IV	0.91	0.51, 1.78	0.772	0.58	0.31, 1.19	0.108
Unkown	2.63	1.48, 5.11	0.002^*^	1.39	0.75, 2.83	0.331
**Laterality**						
Only one side	Ref			Ref		
Both sides	0.65	0.52, 0.79	<0.001^*^	0.49	0.40, 0.61	<0.001^*^
Unkown	3.11	1.87, 4.95	<0.001^*^	1.69	1.10, 2.53	0.013^*^
**Tumor size (mm)**						
≤ 27	Ref			Ref		
≥28	1.48	1.05, 2.18	0.035^*^	2.28	1.54, 3.55	<0.001^*^
**Bone metastases**						
No	–	–	–	Ref		
Yes	–	–	–	1.70	0.57, 4.08	0.280
Unknown	–	–	–	1.27	1.04, 1.56	0.021^*^
**Brain metastases**						
No	–	–	–	Ref		
Yes	–	–	–	4.91	0.68, 25.3	0.067
Unknown	–	–	–	1.27	1.03, 1.55	0.023^*^
**Liver metastases**						
No	–	–	–	Ref		
Yes	–	–	–	1.50	1.08, 2.05	0.013^*^
Unknown	–	–	–	1.44	1.15, 1.80	0.002^*^
**Lung metastases**						
No	–	–	–	Ref		
Yes	–	–	–	1.82	1.30, 2.53	<0.001^*^
Unknown	–	–	–	1.50	1.20, 1.87	<0.001^*^
**CA125**						
Negative	Ref			Ref		
Positive	2.28	1.10, 5.81	0.048^*^	1.07	0.57, 2.31	0.839
Unkown	3.60	1.70, 9.30	0.003^*^	1.63	0.83, 3.58	0.189
**Surgery**						
No surgery	Ref			Ref		
Local resection	0.20	0.09, 0.38	<0.001^*^	0.66	0.38, 1.11	0.126
Debulking or pelvic exenteration	0.07	0.05, 0.09	<0.001^*^	0.13	0.10, 0.17	<0.001^*^
Surgery but the specific operation unknown	0.13	0.01, 0.64	0.046^*^	0.47	0.07, 1.97	0.347
**Radiation**						
No	Ref			Ref		
Yes	0.67	0.16, 1.79	0.495	0.57	0.17, 1.39	0.275
**Chemotherapy**						
No	Ref			Ref		
Yes	0.06	0.05, 0.08	<0.001^*^	0.07	0.06, 0.09	<0.001^*^

FIGO, Federation International of Gynecology and Obstetrics; OR, Odds Ratio; CI, Confidence Interval; Ref, Reference;

* *p < 0.05*.

**Table 3:** The multivariate logistic regression analysis of early death in patients with advanced epithelial ovarian cancer.

**Characteristic**	**FIGO Stage III**			**FIGO Stage IV**		
	**OR**	**95% CI**	***P*-value**	**OR**	**95% CI**	***P*-value**
**Age (years)**						
≤ 63	Ref			Ref		
64–76	2.90	2.17, 3.89	<0.001^*^	1.53	1.14, 2.04	0.004^*^
≥77	4.80	3.44, 6.72	<0.001^*^	2.80	1.96, 3.99	<0.001^*^
**Race**						
White	Ref			Ref		
Black	1.24	0.80, 1.86	0.317	1.12	0.74, 1.66	0.586
Others	0.73	0.42, 1.20	0.234	1.51	0.98, 2.28	0.054
**Marital status**						
Single	Ref			Ref		
Married	0.72	0.52, 1.01	0.051	0.89	0.64, 1.24	0.472
Divorced or Separated	1.18	0.78, 1.78	0.426	0.91	0.58, 1.41	0.664
Widowed	1.10	0.75, 1.62	0.630	0.91	0.60, 1.38	0.657
**Histological subtype**						
Endometrioid carcinoma	Ref			Ref		
Serous cystadenocarcinoma	1.08	0.69, 1.77	0.740	0.37	0.23, 0.58	<0.001^*^
Mucinous cystadenocarcinoma	4.16	2.25, 7.81	<0.001^*^	1.14	0.63, 2.07	0.677
Clear cell adenocarcinoma	3.28	1.79, 6.09	<0.001^*^	0.74	0.38, 1.40	0.351
**Laterality**						
Only one side	Ref			Ref		
Both sides	1.12	0.88, 1.43	0.357	0.94	0.73, 1.22	0.647
Unkown	0.92	0.44, 1.86	0.826	0.51	0.28, 0.90	0.022
**Tumor size (mm)**						
≤ 27	Ref			Ref		
≥28	1.26	0.86, 1.92	0.255	1.44	0.93, 2.33	0.116
**Liver metastases**						
No	–	–	–	Ref		
Yes	–	–	–	1.86	1.26, 2.71	<0.001^*^
Unknown	–	–	–	1.49	0.50, 4.22	0.468
**Lung metastases**						
No	–	–	–	Ref		
Yes	–	–	–	2.19	1.44, 3.29	<0.001^*^
Unknown	–	–	–	1.02	0.36, 3.03	0.967
**CA125**						
Negative	Ref			Ref		
Positive	2.70	1.21, 7.29	0.028^*^	–	–	–
Unkown	2.45	1.07, 6.75	0.054	–	–	–
**Surgery**						
No surgery	Ref			Ref		
Local resection	0.18	0.08, 0.39	<0.001^*^	0.37	0.19, 0.71	0.003^*^
Debulking or pelvic exenteration	0.12	0.08, 0.18	<0.001^*^	0.15	0.11, 0.22	<0.001^*^
Surgery but the specific operation unknown	0.16	0.01, 1.05	0.107	0.39	0.04, 2.25	0.340
**Chemotherapy**						
No	Ref			Ref		
Yes	0.08	0.06, 0.10	<0.001^*^	0.09	0.07, 0.11	<0.001^*^

FIGO, Federation International of Gynecology and Obstetrics; OR, Odds Ratio; CI, Confidence Interval; Ref, Reference;

* *p < 0.05*.

### Construct Nomogram

**Figure 3:**
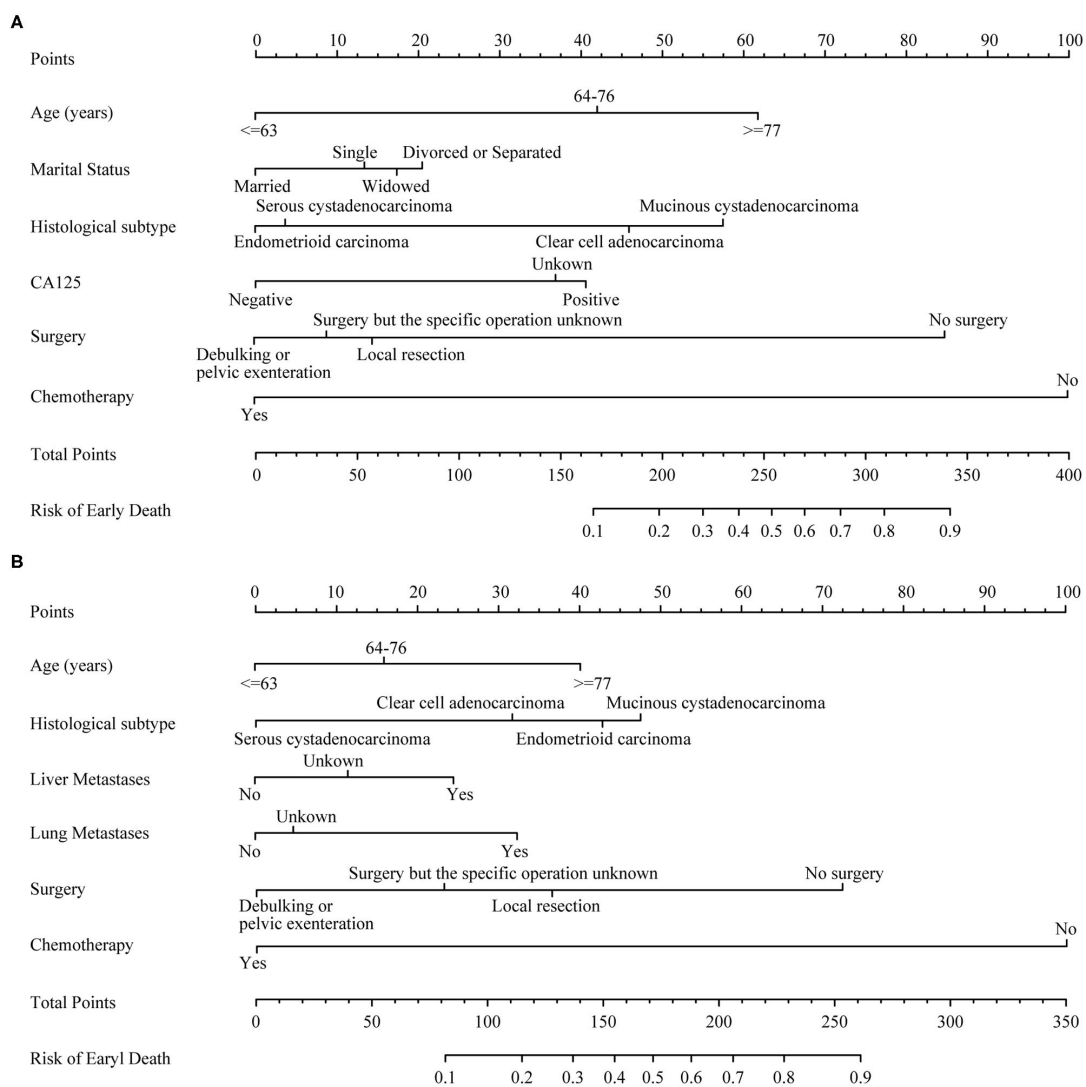
The nomograms of early death in patients with advanced epithelial ovarian cancer. **(A)** FIGO stage III; **(B)** FIGO stage IV.

Several crucial variables from multiple logistic regression were selected, which include age, histological subtypes, liver metastasis, lung metastasis, surgical treatment, and chemotherapy. A nomogram of premature mortality in FIGO stage III and IV ovarian epithelial tumor patients was constructed (Figure 3).

### Performance Evaluation of Nomograms

**Figure 4:**
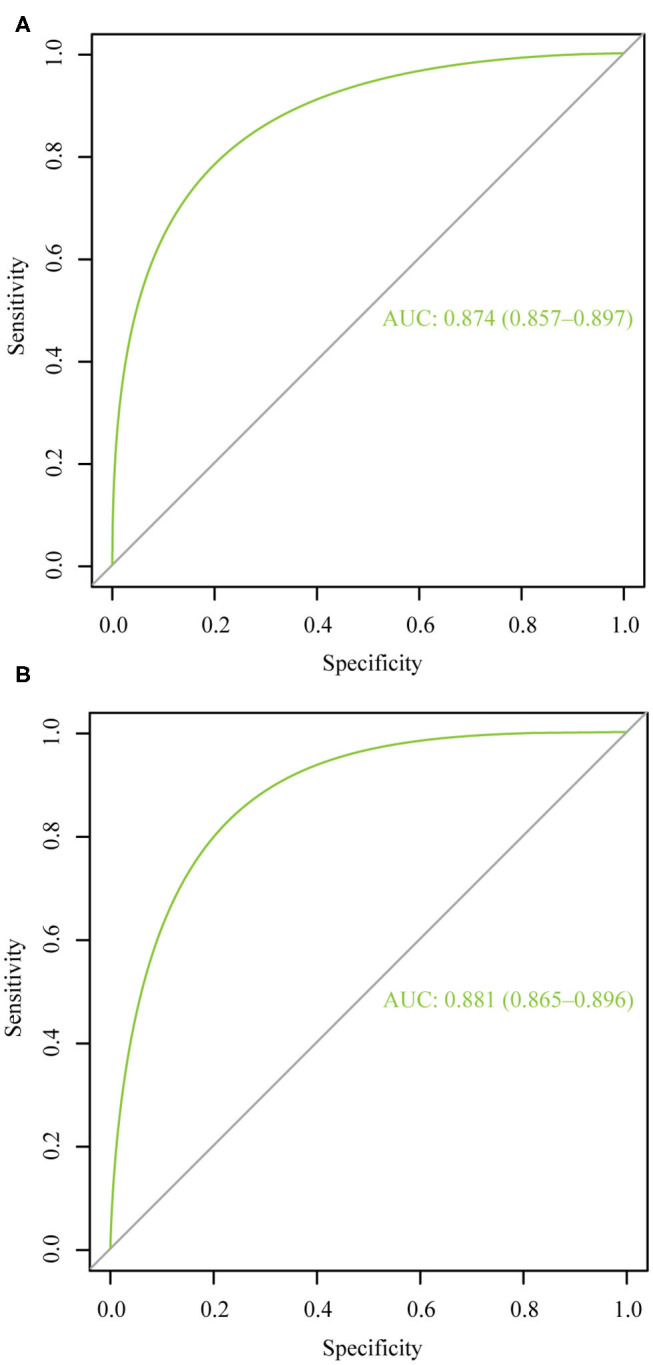
The receiver operating characteristic curve for nomogram. **(A)** FIGO stage III; **(B)** FIGO stage IV. AUC, area under the curve.

**Figure 5:**
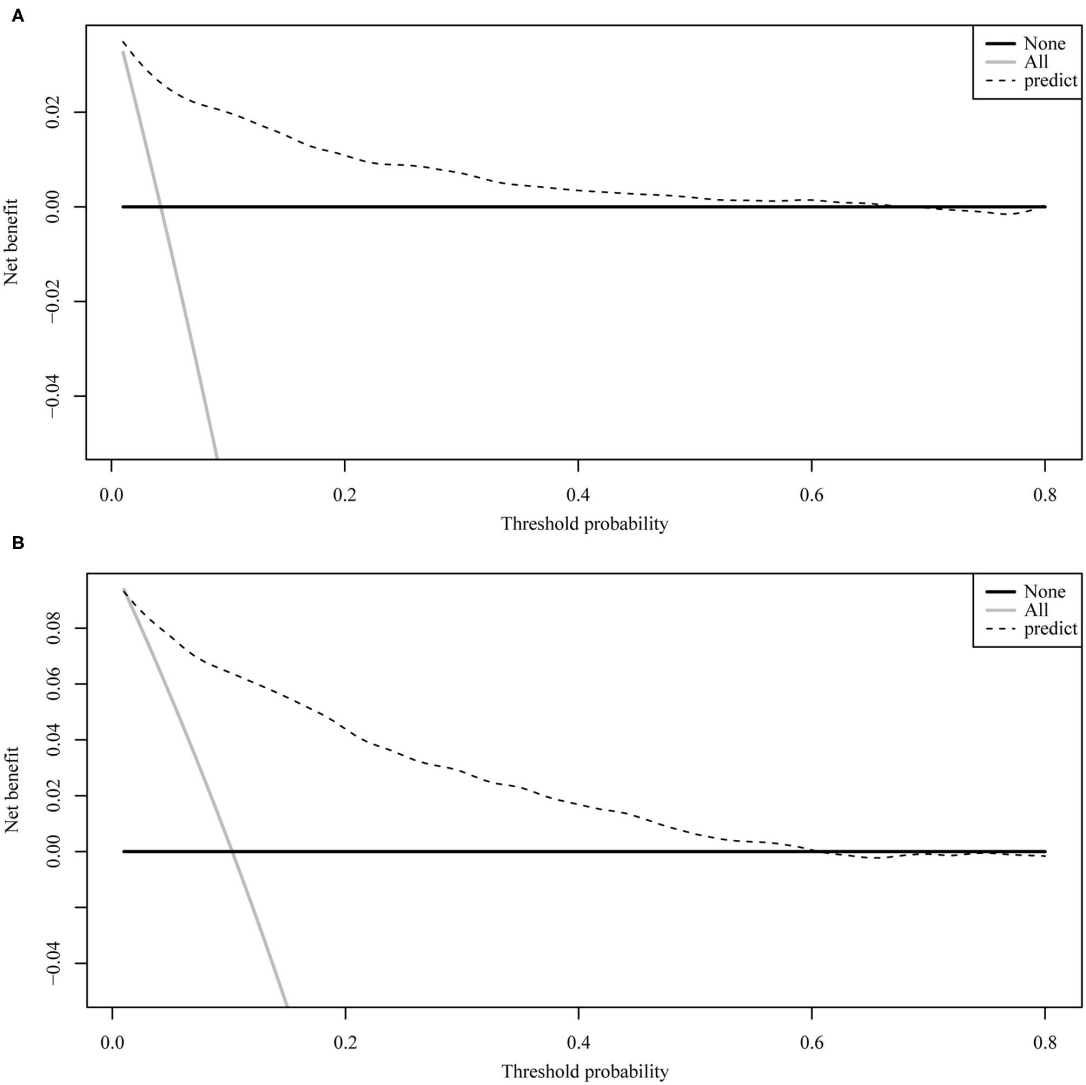
The decision curve analysis curve for nomogram. **(A)** FIGO stage III; **(B)** FIGO stage IV.

**Figure 6:**
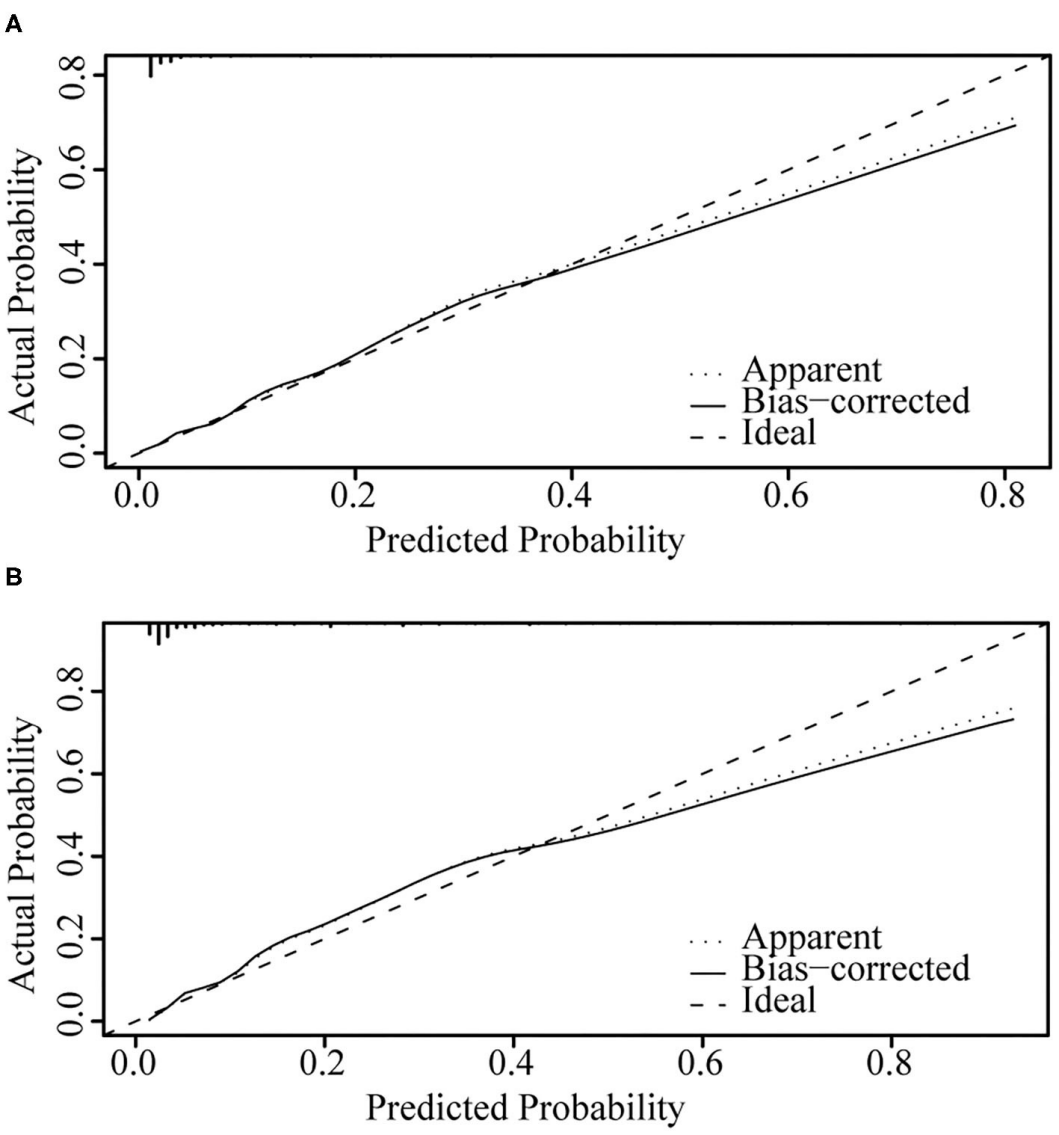
Internal verification plots of nomogram calibration curves by bootstrapping with 1,000 resamples. **(A)** FIGO stage III; **(B)** FIGO stage IV.

Figure 4 illustrates the ROC curve of nomograms used to evaluate FIGO stage III and IV EOC patients. The area under the ROC curve (AUC) of nomograms exceeds 85%, implying that nomograms have satisfactory predictive ability. Moreover, DCA results (Figure 5) show that the predictive model is clinically beneficial. All calibration curves have been internally verified and are close to the 45° line (Figure 6). The C-statistics and Brier scores before and after bootstrapping (1,000 resamplings) are displayed in Table 4. The internal validation demonstrates that the predicted value is consistent with the actual value.

**Table 4:** C-statistic and Breir score of nomograms for the advanced epithelial ovarian cancer.

**Characteristics**	**Nomogram**	**After internal verification**
**C-statistic**		
FIGO Stage III	0.8829	0.8785
FIGO Stage IV	0.8765	0.8723
**Brier score**		
FIGO Stage III	0.0323	0.0327
FIGO Stage IV	0.0689	0.070

## Discussion

Advanced EOC has a higher early death rate. In the present study, the premature mortality rate is 4.23% for FIGO stage III EOC and 10.29% for FIGO stage IV EOC. A set of predictive tools was necessary to identify high-risk patients early death and to provide personalized treatment. A nomogram is a commonly used prognostic tool. In recent years, nomograms have been used extensively to predict the risk and prognosis of malignant tumors (15, 16). Nomograms based on the SEER database have a larger population sample, so nomograms are more accurate and stable (17, 18). The innovation of our study is that, for the first time, the SEER database was used to construct a nomogram to predict the risk of early death in patients with advanced EOC. Our study showed that for FIGO stage III patients, those who were older, divorced/ separated, mucinous cystadenocarcinoma, CA125 positive, and those without surgery or chemotherapy had an elevated risk of premature mortality. For FIGO stage IV patients, those who were more elderly, have mucinous cystadenocarcinoma, liver metastasis, lung metastasis, who had not undergone surgery and chemotherapy have an increased likelihood of premature mortality.

Previous studies have elucidated that age is associated with the prognosis of EOC. Hanatani et al. have shown that younger patients with EOC or borderline EOC under the age of 40 are more likely to survive (19). Cress et al. also showed that younger age was an important predictor of long-term survival in patients with EOC (20). In our study, older age was an important predictor of early death in advanced EOC, consistent with the above findings.

In our study, the histological staging of advanced epithelial ovarian tumors was associated with premature mortality. For FIGO stage III epithelial ovarian tumors, the risk of premature death ranged from low to high for endometrioid carcinoma, serous cystadenocarcinoma, clear cell adenocarcinoma, and mucinous cystadenocarcinoma. For FIGO stage IV epithelial ovarian tumors, the risk of premature death also ranged from low to high for clear cell adenocarcinoma, serous cystadenocarcinoma, mucinous cystadenocarcinoma, and endometrioid carcinoma. Therefore, patients included in the present study with mucinous cystadenocarcinoma had the highest risk of early death, whether they were FIGO stage III or IV epithelial ovarian tumors. Previous studies have shown that mucinous ovarian cancer is usually diagnosed at a low level and early stage, therefore, regardless of the influence of FIGO staging, mucinous ovarian cancer generally has a better prognosis (21, 22). Hess et al. showed that compared with other histological subtypes of EOC, patients with advanced mucinous ovarian cancer had a poorer response to platinum-type first-line chemotherapy and a poorer survival rate (23). Winter et al. studied stage III epithelial ovarian tumors and found that compared with serous tumors, mucinous ovarian cancer and clear cell cancer had inferior progression-free survival (PFS) and overall survival (OS) rate (24). Although it is an epithelial ovarian tumor, the histological molecular structure of the ovarian mucinous tumor is different from other subtypes. Serous tumors are mainly caused by P53 mutations, whereas ovarian mucinous cancer is mainly caused by K-ras mutation (25). With regards to the effectiveness of platinum-based chemotherapy, better results are observed in advanced ovarian serous carcinoma compared with ovarian mucinous carcinoma (26). These may explain why different histological subtypes influence the risk of early death.

CA125 is a specific serum marker used to detect EOC. In the advanced stage of FIGO, CA125 levels are elevated (>35 U/mL) in about 90% of patients (27). However, the effect of CA125 on the prognosis of advanced EOC remains controversial. Studies have shown that PFS and OS are significantly better in advanced FIGO stage EOC when CA125 is <500 U/mL than when CA125 is >500 U/mL (28). However, there are studies that argue against it. Morales-Vasquez et al. described that a high level of CA125 (>500 U/mL) was associated with higher survival rates (29). In our study, for FIGO stage III patients, positive CA125 was associated with early death, while for FIGO stage IV patients, CA125 was not associated with early death. In the SEER database, CA125 only distinguishes between “negative,” “positive” and “unknown.” Unfortunately, due to the limitations of the SEER database, our study was unable to obtain the specific value of CA125 in patients and find the optimal truncation value of CA125 for predicting early death, contributing to one of the major limitations of this study.

The results of the present study are subjected to other limitations. Firstly, as a retrospective study, selection bias was unavoidable. A poor performance status also increase the risk of major complications after the surgery (30). Patients' performance status, comorbidities, Eastern Cooperative Oncology Group (ECOG) score, and other factors were not taken into account, therefore there was also a bias. Secondly, certain factors that might induce premature mortality were not incorporated in the study. For example, EOC has a large number of potential tumor biomarkers, including CA-125, WFDC2 (HE4) protein, and serum mesothelin (31). This study merely included CA125, lacking other important biomarkers. The tumor dissemination can play a role in determining prognosis in ovarian cancer patients (32). Due to the limitations of SEER database, the tumor dissemination, the residual tumor after the surgery, the type of chemotherapy and the maintenance therapy and other information with important significance for the prognosis of EOC were not included in this study, so the above information was not included. Thirdly, the SEER database contains a large amount of unidentified data, which could interfere the results of the model. Moreover, the model was not validated by external clinical data. Future studies could verify the present model with clinical data to assess its credibility.

In conclusion, the nomogram created in this study can effectively predict early death from advanced EOC. Thus, it can help clinicians screen patients at high risk and provide individualized treatment and improved survival.

## Data Availability Statement

Publicly available datasets were analyzed in this study. This data can be found here: https://seer.Cancer.gov/.

## Author Contributions

ZS: research ideas and drafting drafts. YZ: statistical analysis and manuscript writing. XB: data extraction. DZ: conception of research and quality control. All authors contributed to the article and approved the submitted version.

## Conflict of Interest

The authors declare that the research was conducted in the absence of any commercial or financial relationships that could be construed as a potential conflict of interest.
